# Development of High Dose Oseltamivir Phosphate Dry Powder for Inhalation Therapy in Viral Pneumonia

**DOI:** 10.3390/pharmaceutics12121154

**Published:** 2020-11-27

**Authors:** Shahir Aziz, Regina Scherlieβ, Hartwig Steckel

**Affiliations:** 1 Department of Pharmaceutical Technology, Faculty of Pharmacy, German University in Cairo, Cairo 11835, Egypt; 2 Department of Pharmaceutics and Biopharmaceutics, Kiel University, D-24118 Kiel, Germany; rscherliess@pharmazie.uni-kiel.de; 3 Deva Holding A.S., Istanbul 34303, Turkey; Hartwig.steckel@t-online.de

**Keywords:** oseltamivir phosphate, trehalose, alternative carrier, jet mill, dry-powder inhalation, fine diluent, high drug dose, viral pneumonia

## Abstract

Oseltamivir phosphate (OP) is an antiviral drug available only as oral therapy for the treatment of influenza and as a potential treatment option when in combination with other medication in the fight against the corona virus disease (COVID-19) pneumonia. In this study, OP was formulated as a dry powder for inhalation, which allows drug targeting to the site of action and potentially reduces the dose, aiming a more efficient therapy. Binary formulations were based on micronized excipient particles acting like diluents, which were blended with the drug OP. Different excipient types, excipient ratios, and excipient size distributions were prepared and examined. To investigate the feasibility of delivering high doses of OP in a single dose, 1:1, 1:3, and 3:1 drug/diluent blending ratios have been prepared. Subsequently, the aerosolization performance was evaluated for all prepared formulations by cascade impaction using a novel medium-resistance capsule-based inhaler (UNI-Haler). Formulations with micronized trehalose showed relatively excellent aerosolization performance with highest fine-particle doses in comparison to examined lactose, mannitol, and glucose under similar conditions. Focusing on the trehalose-based dry-powder inhalers’ (DPIs) formulations, a physicochemical characterization of extra micronized grade trehalose in relation to the achieved performance in dispersing OP was performed. Additionally, an early indication of inhaled OP safety on lung cells was noted by the viability MTT assay utilizing Calu-3 cells.

## 1. Introduction

Inhalation drug delivery has certainly proven success for targeted lung therapy as in obstructive pulmonary diseases. Furthermore, it has shown great potential in treatment of local lung infections and drug delivery for systemic diseases [1,2]. From the different inhalation delivery systems, Dry powder inhalers (DPIs) are generally breath actuated, and the drug is kept in the dry solid-state phase. DPIs appear to be the most promising devices for future use as they are portable, easy-to-operate, and low-cost devices with superior stability of the formulation over solutions or suspensions [3,4,5].

Only drug particles with an aerodynamic size range of 1–5 µm can pass the lung conducting-zone to reach deep into the respiratory region. These fine particles possess a large surface area with increased cohesive and adhesive properties hindering efficient aerosolization. Furthermore, many inhaled drugs are therapeutically effective in microgram doses, which are minimal amounts to be inhaled alone successfully. Thus, the typical approach to overcome such issues is to blend the micronized drug particles with coarser carrier particles (about 98.5% *w/w*). The bulk carrier powder in such formulations facilitates flowability and dispersion. Traditionally, lactose monohydrate is the main choice as a carrier. It is mass produced from natural sources without toxicity [6,7]. As the drug’s particle-size distribution affects its deposition in the lungs, dispersion of agglomerates to the corresponding individual drug particles used in DPIs is the most important performance characteristic for effective aerosol generation [8]. The adhesion of drug particles to the carrier is governed by four fundamental forces of interaction. Such particle interaction forces are; (a) van der Waals forces from the electromagnetic nature of matter, (b) capillary forces from the presence of water, (c) electrostatic forces arising from the insulating nature of the material, and (d) mechanical interlocking due to surface asperities formed by the production methods [9,10]. The major forces of interaction present a barrier to the particles’ flow and dispersion [11]. Thus, aerosolization performance depends on the deagglomeration power able to overcome such specific interparticular forces of interaction [12]. Intensive studies were conducted on alternative sugars as potential excipients for DPI, showing variation in the behavior of particles’ interaction based on their nature. The studies utilized sugar-based excipients such as dextrose, erythritol, glucose monohydrate, maltitol, maltose, mannitol, sorbitol, trehalose, and xylitol. Those excipients were employed in binary blends as coarse carriers with a particle size range of about 30–200 µm and as fine-sized ternary additives targeting enhanced aerosolization performance [3,10,13,14].

However, this approach is not applicable to effectively deliver high therapeutic doses (>2.5 mg of active inhalable particles) of anti-infective agents per single inhalation shot [15]. High drug doses with higher carrier amount would result in an increased powder volume that is inconveniently bulky for multidose inhalers and may result in several adverse effects as throat irritation and coughing [16]. A trend is to utilize particle engineering techniques such as spray drying to produce carrier-free microcomposite particles with other additives that have low density and special morphology [17]. This approach may deliver better control over the produced particles’ physicochemical characteristics and accommodate high drug loads, yet conventional jet-milling particle preparation technology gives quicker production options with desired surface roughness and crystalline stability that showed superior aerosolization performance in several previous studies [18]. DPI performance is assessed in vitro by impaction methods mainly, evaluating the fine-particle fraction (FPF) aerosolized that can reach the lung. The available therapeutic inhalation products on the market and under research from literature give an FPF range of about 10–80% as collected previously [19]. This shows that there is no optimum formulation/inhaler type or method that can facilitate the successful delivery of all types of drugs, but every drug requires its tailored made formulation.

Oseltamivir phosphate (OP) is the leading drug in the class of oral neuraminidase inhibitors (NAIs), which are the primary treatment option for pandemic influenza and which can also be used to limit the spread of infection for people at high risk, as recommended by the World Health Organization (WHO). OP is a prodrug currently available only as orally administered capsules (Tamiflu™; Roche Laboratories, Nutley, NJ, USA). Oseltamivir is a sialic acid derivative that inhibits the neuraminidase enzymatic active-site specific for influenza virus A and B [20,21,22]. The standard OP treatment regimen is 75 mg twice daily for five days for adults [23]. Recent retrospective clinical studies [24,25] reported the use of OP in combination with other treatment regimens for patients with COVID-19 in the hope of fighting the spread of the new world pandemic. Nevertheless, OP did not show evidence of a direct effect in the treatment of COVID-19, which was related to the absence of viral neuraminidase. Correspondingly, OP in combination therapy with corticosteroids was associated with prolonged viral shedding in several clinical cases that led to prolonged hospitalization. To date, OP is considered the first line of treatment in pandemic influenza and used with caution as a protective measure from secondary influenza infections in other pneumonia cases [26,27]. Indeed, it may help to minimize the burden of the healthcare system in the current pandemic due to severely affected patients with double-infection cases with influenza and SARS-CoV2.

Inhalation administration of OP targets the virus at the infection site instead of the drug’s systemic administration. This concept is similar to aerosolized zanamivir, another NAI being topically administered as a DPI with a daily dose up to 20 mg [28]. From marketed Relenza^®^, one blister contains 5 mg of zanamivir formulated with 20 mg lactose monohydrate as a carrier [29]. It is hypothesized that delivering OP directly into the lungs by inhalation would lower the dose, lower the oral side effects (i.e., nausea and vomiting), and give direct faster activity. Although OP requires metabolic activation, the lung that is the site of direct administration is rich in carboxylesterase (CES 1) enzyme [30]; hence, the drug can be activated at the site of action. Further results discussed in a study testing the metabolism of OP in rat lung showed successful transformation into the active form [31]. In that study, they prepared oseltamivir phosphate liposomes as inhalation powder by spray drying reaching an average of 35.4% fine-particle fraction when evaluated for aerodynamic performance. Another recent study had worked on developing OP in a sustained release formulation reaching a maximum of 1.08 mg fine-sized particle dose as tested by impaction for aerodynamic performance [32]. Although their achieved dose might not be high enough to be effective in a single inhalation shot, they showed the importance of developing the antiviral drug in a pulmonary targeted formulation.

This study aimed at delivering high-dose OP in a DPI formulation using safe and conventional sugars as diluents. The second key objective was the optimization of aerosolization performance of the high OP dose based on the diluent particle size and OP/diluent ratio. In a comparative approach, different binary blends of micronized OP with a milled or micronized diluent were prepared. Type, amount, and particle size of diluents were varied to assess and compare their aerosolization performance. The particle-size distribution (PSD) of both of the drug and the diluent excipient and the delivered dose of a pharmaceutical aerosol is significant in the development of formulations for pulmonary delivery and in the quality control of pharmaceutical aerosols. The assessment was done by cascade impaction using the next generation impactor described in the Pharmacopoeia for the characterization of the PSD of aerosols. A newly developed inhaler with medium resistance (UNI-Haler) [33] was used as the inhaler for aerosolization tests evaluating its usefulness in OP delivery. Further in vitro characterization of the prepared extra fine particulate formulations based on trehalose diluent was performed to confirm and understand their resulting behavior.

## 2. Materials and Methods

### 2.1. Materials

OP was kindly provided by the Nile Co. for Pharmaceuticals and Chemical Industries, Cairo, Egypt. Trehalose (coarse grade) was provided by Cargill Deutschland GmbH (Krefeld, Germany). Mannitol 60 (coarse grade) and glucose monohydrate (coarse grade) were provided from Roquette, Ludwigshafen, Germany. Lactose inhalation fine grades were of two types; Respitose^®^ ML006 was kindly provided by DMV-Fronterra Excipients, Nörten-Hardenberg, Germany, while Lactohale^®^ LH 300 was kindly provided by Friesland Foods Domo, Borculo, The Netherlands. Calu-3 (HTB-55) cells were purchased from the American type culture collection (ATCC, Manassas, VA, USA). MTT (3-(4,5-dimethylthiazol-2-yl-2,5-diphenyltetrazolium bromide), Hank’s buffered salt solution (HBSS), HEPES (4-(2-hydroxyethyl)-1-piperazine-ethanesulfonic acid) buffer solution, minimum essential medium (MEM) with Earle’s salt, fetal bovine serum, penicillin-streptomycin, nonessential amino acids, and sodium pyruvate were all supplied from Biochrom AG, Berlin, Germany. All reagents (Brij35, ethanol, glycerol, sodium dodecyl sulfate (SDS), dimethylformamide (DMF), trypsin, and Ethylenediaminetetraacetic acid (EDTA) used were of analytical grade and supplied by Merck KGaA (Darmstadt, Germany).

### 2.2. Powder Preparation

#### 2.2.1. Micronization

OP, trehalose, mannitol, and glucose batches were micronized by a fluid energy jet mill Jet-O-Mizer (Aljet mill, Fluid Energy processing and equipment company, Plumsteadville, PA, USA). Target particle size (x50 ≤ 5 µm) was controlled by two variables: grinding pressures (5–10 bars) and number of runs (1–2 runs). The starting material was weighed in a 10-g sample for each micronization run and inserted into the feed hopper. The material was conveyed into the milling chamber by the high-pressure pushing nozzle where the pusher nozzle was set one bar higher than the grinding nozzle. A design of experiments (DoE) was carried out to evaluate the effect of feed variables on the trehalose particle size produced by jet milling. D-optimal design was conducted and evaluated by Design-Expert^®^ software version 8.

#### 2.2.2. Milling

A rotor-speed mill (Pulverisette 14 model 14.102/2304, Fritsch GmbH, Idar-Oberstein, Germany) was used to reach larger target particle size (x50 ≥ 15 µm). For the milled grade batches, trehalose, mannitol, and glucose a 15-g sample of each was fed into the grinding rotor through a funnel and then passed through a 0.2 mm sieve ring into a collector pan. A similar target particle size for each was controlled by two variables: grinding rotational speed (16,000–20,000 rpm) and number of runs (1–2 runs).

#### 2.2.3. Particle-Size Distribution

For particle-size distribution analysis using Helos-Laser diffraction, a powder sample was dispersed in a RODOS module at 3 bars (Sympatec GmbH, Clausthal-Zellerfeld, Germany). The measurements were triggered at an optical signal of 0.5% and stopped after 5 s of real-time measurements. Particle size distribution calculation was based on the Fraunhofer light diffraction theory, as previously described [34]. As all actual nonspherical particles measured for their corresponding diameter of a sphere, enhanced precision was achieved by using different optical modules; R1 lens with focal length (*F*) *=* 20 mm to accommodate 0.1–35 µm particles or R2 lens (*F* = 50 mm) to accommodate larger particles up to 87.5 µm. The measurements were performed in triplicate and data acquisition and calculations made with Helos-Sympatec Window X5 (version 5.4.2.2) software.

#### 2.2.4. Blending OP with Different Excipients

Targeting a high-dose formulation and examining the effect of the diluent nature and particle size, OP was mixed with four types of diluents with two different particles size grades (milled and micronized) in the ratio of 1:1. To test the effect of mixing ratios, further formulations were prepared of micronized OP with fine trehalose at 1:3 and 3:1 weight ratios. To have a deeper insight on the effect of fine diluent particles, additional formulations of micronized OP with different micronized trehalose grades were prepared in a fixed ratio as shown in Table 1.

All the binary DPI formulations were prepared first by mixing, and sieving, followed by two blending runs in a 30 cm^3^ stainless steel vessel in a Turbula Mixer (Turbula T2C, Willy A Bachofen AG, Basel, Switzerland). The diluent geometric mixing with OP gave an equal primary distribution of the unequal amounts before blending. The mixed powder was then passed through a 355 µm sieve to disrupt large agglomerates and give a flowing powder. The powder was then blended using the Turbula Mixer at 42 rpm for 10 min. After first blending, a second sieving step was performed, and the blending was repeated under the same run conditions and then passed through the sieve again for breaking down large agglomerates. Afterwards, drug distribution and homogeneity among each powder blend were evaluated. For this, three samples top, middle, and bottom were taken from each formulation dissolved in bidistilled water, and drug concentration was assessed using a standardized spectrophotometric assay (2.2.6). To prevent the changing environmental factors from altering the particle’s interaction behavior from batch to batch, the whole preparations were done in an environmental controlled chamber at relative humidity (RH) below 40% and temperature below 25 °C.

#### 2.2.5. UV-Spectrophotometric Quantification

Quantification of OP was performed by UV-spectrophotometric analysis at a wavelength of 240 nm. Samples were analyzed by double beam UV/Vis spectrometer (Lambda 40, PerkinElmer^®^, Waltham, MA, USA). The method has been validated in terms of linearity, assay precision, and accuracy by different concentration sets of pure OP and OP in combination with diluent. The linear concentration range tested was 0.0125, 0.025, 0.05, 0.1, and 0.2 mg/mL. Lactose, trehalose, mannitol, and glucose had no significant absorbance at 240 nm, and OP solutions were prepared by bidistilled water; accordingly, the quantification of OP was simple and sensitive for the tested concentrations.

### 2.3. Trehalose Physicochemical Properties

#### 2.3.1. X-ray Powder Diffraction

X-ray powder diffraction was used to evaluate the integrity of trehalose particles’ crystalline structure before and after micronization. Samples were analyzed by an X-ray diffractometer with a rotating anode (Stoe and Cie GmbH, Darmstadt, Germany) with a Cu-K_α_ source operated at 40 kV and 30 mA.

#### 2.3.2. Specific Surface Area

A gas sorption analyzer (NOVA 2200, Quantachrome Corporation, Boynton Beach, FL, USA) was used to measure the specific surface areas of the micronized trehalose particles based on the Brunauer–Emmett–Teller (BET) theory. Samples were conditioned overnight under vacuum at room temperature before analysis by an eleven-point nitrogen adsorption BET isotherm at 77 K was performed in triplicate. 

#### 2.3.3. Fluidization energy

An FT4 powder rheometer (Freeman Technology, Gloucestershire, UK) was utilized to measure the fluidization energy of different micronized trehalose grades under aerated conditions. For each run, a fixed volume of 25 mL powder sample was examined in a 30 mm bore borosilicate glass cylinder. The powders were assessed using an automated aeration module that runs a sequence of tests to determine the response at increasing levels of air velocity through the powder sample (0–10 mms^−1^), with a conditioning cycle before each test cycle. The fluidization energy is determined when the flow energy plateaus at increasing air flow velocities [35].

#### 2.3.4. Scanning Electron Microscope

Scanning electron microscopy (SEM, Carl Zeiss Microscopy GmbH, Oberkochen, Germany) was used to investigate the morphological features of micronized trehalose and OP blend. Samples were mounted on sticky carbon tabs and sputtered with gold using a BAL-TEC SCP 050 Sputter Coater (Leica Instruments, Wetzlar, Germany) before imaging.

### 2.4. Aerosolization Performance of OP/Diluent Blends

**Figure 1:**
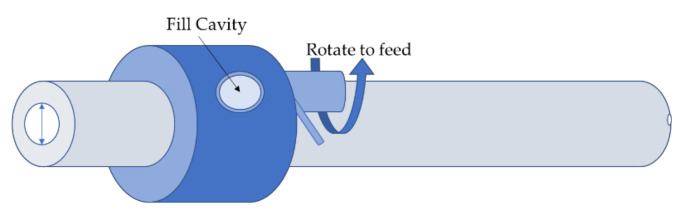
Schematic diagram of the Rack showing the fill cavity and the rotation mechanism to feed into the tube adopted with permission from [14], Elsevier, 2004.

A next generation impactor (NGI, Copley Scientific, Nottingham, UK) was used for evaluating the aerodynamic particle-size distribution for each prepared formulation to assess the aerosolization performance following United States Pharmacopoeia (USP) Chapter 601 [36]. For testing via the capsule-based inhaler, each formulation blend was accurately weighed as 15 ± 0.1 mg filled sample into size three hydroxypropyl-methylcellulose (HPMC) capsules (Quali-V^®^ caps, Qualicaps^®^ Europe, S.A.U., Madrid, Spain). To prevent powder particles from bouncing, NGI preseparator and tray cups were precoated with a solution of Brij35:ethanol:glycerol at a weight ratio of 15:51:34, respectively. Two methods of aerosolization were adopted for each blend of the different diluents. The first one was performed without an inhaler as a control, using an application device called “Rack” instead of the inhalation device to eliminate the influence of the inhaler and allow a low-turbulent flow of the air with no dispersion aids such as a swirl chamber or baffles. Hence, this would measure the aerosolization performance depending only on the incomplete dispersion nature of the powder particles under a low-turbulent air flow. Briefly, the “Rack” (Figure 1) consists of a stainless-steel tube with an inner diameter of 5 mm and a total length of 170 mm [14]. For the delivery of the powder into the air stream, powder was weighed (15 ± 0.1 mg) into the cavity, released into the stainless-steel tubing by rotating the inner part of the applicator, and entrained by the air, and then the powder was discharged into the NGI from the rack at 80 L/min for 3 s. Prior to the aerodynamic performance examination, flowrate determination of the inhaler devices was done to reach a pressure drop of 4 kPa as noted by the USP specifications.

**Figure 2:**
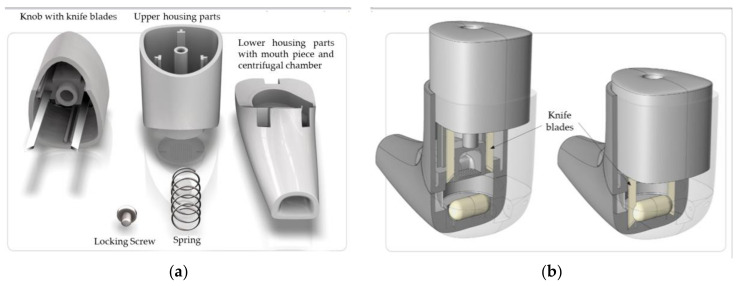
(**a**) Different parts of the UNI-Haler and (**b**) assembly and functional principle of the UNI-Haler.

In the second method, the UNI-Haler was used as the inhalation device representing a medium flow resistance inhaler. Its design is based on simplicity and ease of use. Its functional principal gives a reliable opening of the capsule with two blades with no capsule debris (Figure 2).

The capsules discharged into the NGI by the UNI-Haler at 56.5 L/min for 4.3 s similarly to reach a pressure drop of 4 kPa. In addition, blends with fine trehalose were also discharged by the capsule-based commercial low-resistance inhaler Aerolizer^®^. This additional trial was performed to investigate the behavior of the formulations at 4 kPa pressure drop using the model low-resistant inhaler, which is operated at 100 L/min airflow in comparison to the medium-resistant UNI-Haler, which reaches a pressure drop of 4 kPa at 56 L/min airflow. For each experimental run, one capsule being filled with the respective formulation was used to be discharged into the NGI. Afterwards, the NGI apparatus was disassembled and the inhaler, the throat, the preseparator, and tray cups were rinsed with known volumes of bidistilled water. The amount of OP drug deposited in each part was determined by the spectrophotometric assay. All formulations were assessed in triplicate in an environmentally controlled chamber at relative humidity (RH) of 40% and temperature below 25 °C.

The Copley software was used to calculate the fine-particle dose (FPD), the fine-particle fraction (FPF), and the mass median aerodynamic diameter (MMAD) from the emitted dose. Statistical analysis of data was performed using the GraphPad Prism 6 (GraphPad Prism windows 6.07) (GraphPad Software Inc., San Diego, CA, USA), applying three-way ANOVA, and the results were considered significantly different if *p* values were < 0.05.

### 2.5. MTT Assay for Cytotoxicity Testing on Bronchial Adenocarcinoma Cells

The toxicity of pure micronized OP alone (OP-100%) and with 50% micronized trehalose (OP/TR-50%) was assessed using the MTT (3-(4,5-dimethylthiazol-2-yl-2,5-diphenyltetrazolium bromide) assay (Mossmann 1983). The method adopted from [37] uses the Calu-3 cell line derived from a bronchial adenocarcinoma as a suitable model for the respiratory tract. Two stock solutions of 0.4 mg/mL were prepared for each of pure OP and T1-50 formula by dissolving a 4 mg sample in a 10 mL HBSS with HEPES buffer solution. In reaction caps, nine concentrations covering expected inhaled amounts were then prepared for each by diluting an amount of 0.4 mg/mL stock to 1000 µL by HBSS + HEPES in a 1:1 ratio to contain 0.025, 0.05, 0.1, 0.15, 0.2, 0.25, 0.3, 0.35, and 0.4 mg/mL.

From the subcultivation, the medium was removed from the monolayer cells grown in 96-well plates by gentle sucking and the sample solutions were added and incubated for 4 h at 37 °C. Subsequently, the sample solutions were removed and 200 µL of MTT solutions (5 mg/mL in HBSS + HEPES) was added to each well. Then, the cells were incubated for 2 h. Finally, all cells were lysed with 100 µL lyse solution (5% SDS in DMF: water 50:50, pH 4.7) per well; a solution of 5 mM SDS in HBSS + HEPES was used as a positive control (0% viability), while HBSS + HEPES served as a negative control (100% viability). Detection and quantification of the formazan crystals were performed by the multiwall plate reader at 570 nm with reference wavelength 690 nm (Thermo Spectra III Reader with software easy-WIN-fitting, V6.0a, Tecan, Austria).

## 3. Results and Discussion

### 3.1. Prepared Powder Particles

Carrier physicochemical characteristics are essential for a successful and efficient aerosolization process. Such properties comprise particle-size distribution, specific surface area, surface energy, morphology, and fluidization energy. Although lactose is the most commonly used carrier in inhalation products, it shows limitations with certain drugs. Each of the chosen alternative sugar carriers has shown certain advantages over lactose in DPIs in previous studies [38]. In addition, mannitol and trehalose are nonreducing sugars/sugar alcohols unlike lactose, which interacts with certain amino functional groups by the Maillard reaction. Such chemical degradation interactions by lactose were observed in low molecular weight drugs as formoterol [3] and in protein-based drugs [39]. As for the choice of the glucose, it was previously used in a successful marketed product (Bronchodual^®^) in France by Boehringer Ingelheim. Other sugar alcohols such as xylitol and sorbitol were not included in this study due to their well-known high hygroscopicity that diminishes the deagglomeration efficiency of the dry powder [10].

It is essential for best performance of OP DPI formulations that the vast majority of OP particles are below 5 µm whereas for carriers, normally, a much larger particle size than the active ingredient is selected [40,41]. As described in the literature [42,43], jet milling is the most useful and traditional technique for particle-size reduction to reach the low micrometer range (≤5 µm), although there is no control over the produced particle morphology [10,44]. The unconventional approach of the current study was to formulate binary blends of micronized OP with fine diluent particles that are only slightly larger than or of similar size distribution as the active pharmaceutical ingredient (API). As shown in Table 1, each diluent was reduced in size into a micronized (x50 ≤ 5 µm) and a milled (x50 ≥ 15 µm) quality to enable the investigation of the effect of diluent particle size on the performance. OP was treated for micronized grade only and jet milled by two runs at 10 bars. OP particles size distributions (x10, x50, and x90) that were obtained were 0.58 ± 0.045 µm, 1.61 ± 0.12 µm, and 6.22 ± 0.53 µm, respectively. Lactose was already supplied in the desired size specification and did not need further treatment. It was observed that glucose (G1) needed the highest force to break down its particles and the x90 (21.32 ± 0.67 µm) for the micronized grade was notably higher than the other excipients (T1 and M1) under the same micronization runs and pressure. This may indicate a strong crystal structure of glucose formed in response to its relatively higher hygroscopicity. Trehalose, in specific, was under the spotlight of the analysis and characterization testing, particularly as blends with trehalose had shown high efficiency in dispersing OP in the aerosolization assessment compared to the other excipients.

The design of carriers with relatively large surface area, having microscopic or nanoscale surface roughness, was found to be desirable for aerosolization performance [45,46]. BET gas adsorption allows assessment of SSA of the bulk sample with all different size classes present and will thus give the total area available for particle–particle interactions. From studies on conventional carrier formulations, the higher the surface area, the higher the particle adherence and thus a possibly negative effect on powder aerosolization. So, in addition to the milled and micronized grades, trehalose particles were prepared in extra fine grades to further understand the significance of small variations in the particle-size distribution, surface area, and fluidization energy of the diluent particles on the aerosolization performance. From the factorial design (D-optimal model by Design-Expert^®^ software, version 8.0) for the micronization process, three trehalose grades (T5, T1, and T11) were chosen representing large, medium, and small mean particle-size diameter. The three different batches of jet milled trehalose were successfully produced employing different grinding conditions. The batches were further evaluated for fluidization energy by FT4, specific surface area by the BET gas sorption analyzer, and particle morphology by SEM. The three fine batches were evaluated for their effect on the aerosolization performance in medium-and low-resistance inhaler devices. As anticipated, the finer the trehalose particles, the higher the surface area and the higher the fluidization energy (as the results shown in Table 2).

In target of delivering relatively high drug doses to the lungs in milligrams rather than the conventional microgram doses, different strategies were utilized in previous studies, either by developing new inhalers, by inhaling multiple doses [47], or by developing a dry-powder formulation that accommodates higher drug doses [29,48]. In this study, the amount of powder per dose (including diluent) was set to 15 mg. In addition, one inhalation dose of Relenza^®^ contains 5 mg of zanamivir, which is equipotent with oseltamivir [49]. Accordingly, 15 mg total powder formulation per capsule is estimated to produce a similar dose range for OP. From here, 1:1 blends of OP and each diluent were prepared and tested for aerosolization performance. To test the effect of mixing ratios, further formulations were prepared of micronized OP with fine trehalose at a 1:3 and 3:1 weight ratio. All DPI blends prepared were considered homogenous as their relative standard deviation (RSD) was below 6% [50].

### 3.2. Effect of Micronization on Trehalose Physicochemical Characteristics

#### 3.2.1. Micronized Trehalose Crystalline Structure

**Figure 3:**
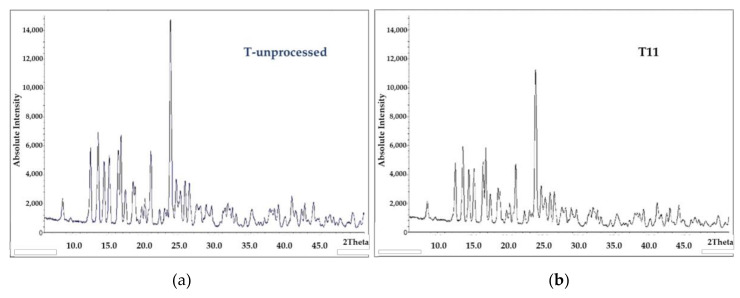
XRPD spectra of trehalose di-hydrate (**a**) before micronization (T-unprocessed) and (**b**) after the finest micronization (T11).

As shown in Figure 3, XRPD spectra showed identical diffraction patterns for trehalose dihydrate before and after micronization, and they are similar to those in the literature [51,52]. This observation confirms the preservation of crystalline structure integrity of the trehalose particles even after micronization. Nevertheless, their detected intensity was slightly reduced upon increasing stress of the micronization conditions indicating the formation of some amorphous content. The intense mechanical processing constantly affects the crystallinity of the material. Based upon the theory of active sites, amorphous particles produced would have high energy active sites that bind strongly to the drug particles reducing the overall aerosolization performance [53]. However, the impact of such amorphous sites would be significant especially in low-dose drug formulations with conventional larger diluent particles. Furthermore, excipient fine particles can act in collaboration with the drug particles forming agglomerates that may deliver the drug by itself acting as fine diluent particles [54]. With regard to the high dose used in this study and fine excipient diluent, the amount of OP particles is way above the saturation limit of such active sites. Additionally, due to particle-size similarity, OP particles possibly will not have the accessibility to any active sites on the fine excipients’ particles. On the other hand, OP particles may have adsorption contact to highly active points on the surface of the fine excipient particles forming agglomerates to be delivered.

#### 3.2.2. Micronized Trehalose Morphology and Orientation within Blends

**Figure 4:**
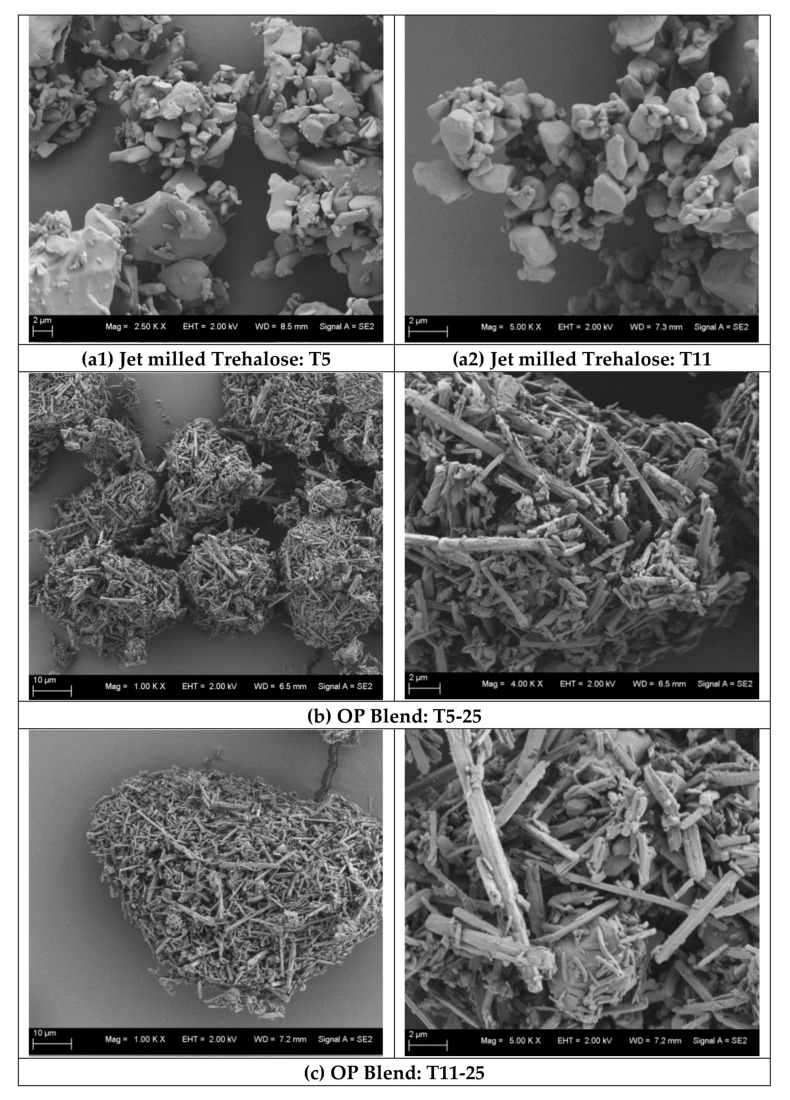
SEM images of (**a**) jet milled trehalose qualities (**a1**) T5 (×2.5 k) and (**a2**) T11 (×5.0 k), (**b**) OP/T5 blend (left: ×1.0 k and right: ×4.0 k) and (**c**) OP/T11 blend (left: ×1.0 k and right: ×5.0 k).

Changing particle surface properties have an influence on the performance variation between different produced batches. In general, jet milling leads to surface heterogeneity of particles implying a nonhomogeneous energy distribution and typically results in irregular morphology. Trehalose particles of the produced small (T11) and large (T5) qualities showed similar morphology as depicted by SEM images in Figure 4. Thus, the main difference between T5 and T11 is the particle size as previously evaluated by laser diffraction. Both qualities were captured as pure material and in blend formulations with OP. As observed, jet milled trehalose qualities were of irregular shape while the OP had rod-shaped morphology. When the relatively cohesive OP particles were blended with T5, the rod-shaped particles were totally covering and surrounding larger particles of trehalose in a separate agglomerated pattern (i.e., formed relatively smaller agglomerates). However, when the OP rod particles were in formulation with the fine T11, a more homogeneous phase of agglomerated rods with irregular trehalose particles imbedded within was observed (i.e., relatively larger agglomerates). It is also observed that OP rod particles are not in complete attachment to the trehalose particles’ surface, but in contact with or adsorbed at their active surface points. Accordingly, not following the active site theory as previously noted, it can be assumed that the trehalose fine particles used did not act as regular carrier on which the drug particles are fixed, but the fine trehalose particles can be considered as spacer particles embedded within a mesh of cohesive drug particles.

### 3.3. Aerosolization Performance of 1:1 Formulations

**Figure 5:**
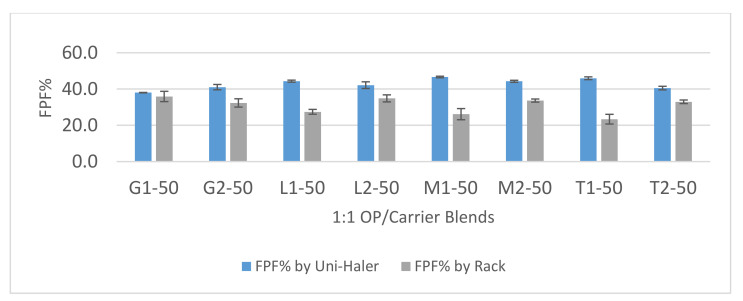
Fine-particle fraction (FPF)% chart of 1:1 OP/diluent blends aerosolized by the UNI-haler and the Rack. Blends are arranged according to diluent grade from left to right; micronized glucose (G1), milled glucose (G2), micronized lactose (L1), milled lactose (L2), micronized mannitol (M1), milled mannitol (M2), micronized trehalose (T1), and milled trehalose (T2). Error bars based on calculated standard deviation from mean (*n* = 3).

**Figure 6:**
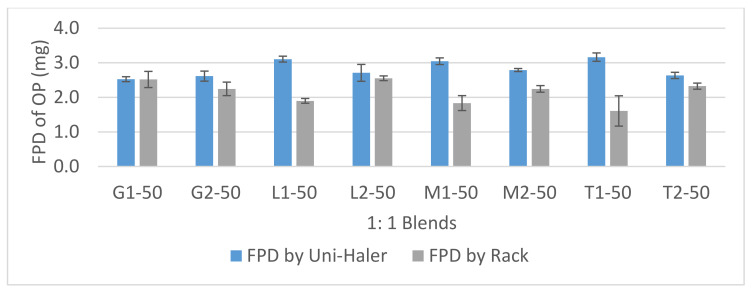
Fine-particle dose (FPD; mg) chart of 1:1 OP/diluent blends aerosolized by the UNI-haler and the Rack. Blends are arranged according to the diluent grade from left to right; micronized glucose (G1), milled glucose (G2), micronized lactose (L1), milled lactose (L2), micronized mannitol (M1), milled mannitol (M2), micronized trehalose (T1), and milled trehalose (T2). Error bars based on calculated standard deviation from the mean (*n* = 3).

The aerosolization performance of the 1:1 blends, as indicated by the FPF% below 5 µm, is shown in Figure 5 and the fine-particle doses of OP are shown in Figure 6. During aerosolization testing, negligible device and capsule retention was observed and thus the delivered dose was similar to the loaded dose (7.5 mg). The comparison was done between the performance of milled and micronized grades of the four diluent materials and the performance of the UNI-Haler against the Rack, which was used as a control eliminating the influence of the inhaler on blend deagglomeration and dispersion. The low-turbulent flow of air through the rack tube without any deagglomerating mesh would measure the aerosolization performance depending only on the physical nature of the powder particles. From the results using the Rack, blends with milled grade diluent showed generally a higher FPF than their corresponding micronized grade blends. Blends based on milled diluents, having relatively larger particles, were easier to partially deagglomerate due to their lower cohesive/adhesive interactions probably being governed by less contact points between the particles. The ANOVA statistical model was used to compare the effect of three sources of variation (independent variables); carrier type, carrier size and inhaler device on the aerosolization performance of 1:1 blend based on the FPF (continuous dependent variable). Tukey’s multiple comparisons post-hoc test was employed to determine the differences in effect lying between the various groups. From Table 3, the sum of squares for each is divided by the degrees of freedom to compare ratios and determine whether each variable has a significant difference either individually or combined on the FPF percentage value. By statistical comparison between micronized and milled grades for both lactose and mannitol, they showed a mean difference in FPF percentage value of (−7.36 and −7.46) respectively. Besides, trehalose showed the highest mean difference (−9.57) in FPF percentage value between its micronized and milled grades under the flow properties of the rack. This was not the case for the glucose blends, where the micronized grade showed insignificant difference (*p* value = 0.43) with the milled grade. This may indicate a high adhesion/cohesion power for trehalose compared to the other sugars tested.

Dispersion using the UNI-Haler increased significantly the FPF and FPD values for all the 1:1 formulations compared to the rack except for the milled grade glucose (*p* value = 0.9695). The general increase in the aerosolization performance indicates the good deagglomeration power of the UNI-Haler and its ability to disperse even highly agglomerated particles to inhalable fine particles. Further, micronized grade diluents showed higher deagglomeration resulting in higher FPF than their corresponding milled grade when aerosolized by the UNI-Haler. For lactose, mannitol, and glucose, the increase in FPF% between micronized and milled grades was not significant with *p* values of >0.05. However, then, again, trehalose micronized grade showed a significant increase in FPF% compared to its milled grade (*p* value = 0.0286).

The results established that T1-50 gave very good aerodynamic particle dispersion using the UNI-Haler with FPF (46%) and FPD (3.2 mg). Similarly, this was followed by the micronized mannitol blend (M1-50) and micronized lactose blend (L1-50) with FPF of 47.3% and 44.3%, respectively, and FPD of 3 mg and 3.1 mg, respectively. Furthermore, the micronized trehalose blend (T1-50) has recorded the highest mean difference of 22.53% in FPF compared to the other diluents when aerosolized by UNI-Haler versus the rack. This indicates that micronized trehalose particles had the ability to form the most clustered agglomerates with OP giving the largest mean dispersed size from the rack (MMAD of 3.75 µm), yet, upon the application of sufficient deagglomeration force by UNI-Haler, it dispersed into the finest mean size (MMAD of 2.8 µm). Adding to the search for an optimized formulation performance, powder flowability is a major issue facing prepared dry formulations. Intensive effort is spent to obtain a free-flowing powder to avoid issues such as weight variation in filling capsules, poor content uniformity, and bad reproducibility [55]. Therefore, it is of great importance to balance between strong agglomerates that would give free-flowing properties and the easiness of deagglomeration upon aerosolization for efficient deposition. Although micronized trehalose, lactose, and mannitol showed insignificant differences regarding their aerosolization performance for OP, fine trehalose particles could form slightly better structured agglomerates with OP that may lead to the required dispersion properties with the highest fine-particle dose. From this first assessment, another question was raised: is it possible to maximize the delivered dose of OP by using a higher drug to excipient ratio within the formulation? Or would the high cohesiveness of the drug particles lead to poor deagglomeration behavior at higher concentrations? Accordingly, a further set of experiments was conducted to show how physicochemical characteristics and a blend ratio of micronized grade trehalose influence the performance in dispersing OP.

### 3.4. Aerosolization Performance of OP/Trehalose Formulations

#### 3.4.1. Dispersion of 3:1, 1:1, and 1:3 OP/Trehalose Formulations

**Figure 7:**
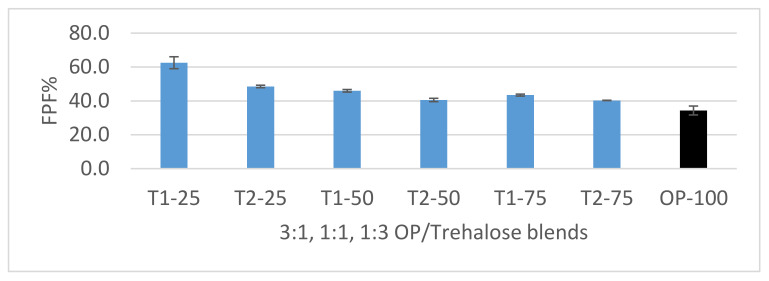
FPF% chart of 3:1, 1:1, and 1:3 OP/trehalose blends and diluent free formulation (OP-100) (black bar) aerosolized by the UNI-haler. Blends (blue bars) are arranged according to trehalose grade and ratio from left to right; 25% micronized trehalose (T1-25), 25% milled trehalose (T2-25), 50% micronized trehalose (T1-50), 50% milled trehalose (T2-50), 75% micronized trehalose (T1-75), and 75% milled trehalose (T2-75). Error bars based on calculated standard deviation from mean (*n* = 3).

**Figure 8:**
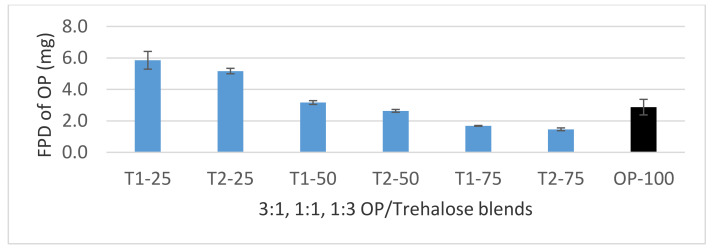
FPD chart of 3:1, 1:1, and 1:3 OP/trehalose blends and diluent free formulation (OP-100) (black bar) aerosolized by the UNI-haler. Blends (blue bars) are arranged according to the trehalose grade and ratio from left to right; 25% micronized trehalose (T1-25), 25% milled trehalose (T2-25), 50% micronized trehalose (T1-50), 50% milled trehalose (T2-50), 75% micronized trehalose (T1-75), and 75% milled trehalose (T2-75). Error bars based on calculated standard deviation from mean (*n* = 3).

The different blend ratios FPF% below 5 µm are shown in Figure 7 and the fine-particle doses of OP are shown in Figure 8. Maximum FPF of 62.5% utilizing the UNI-Haler was reached by the formulation with 25% micronized trehalose (T1-25) and a minimum FPF of 40.3% by the 75% milled trehalose (T2-75). The assessment was done in comparison to a diluent free formulation (OP-100), which showed highest agglomeration and hence lowest FPF of 34.3%. OP particles alone showed high cohesiveness that restricted the ability of the inhaler to deagglomerate the micronized API effectively upon aerosolization. Considering these results, the diluent free formulation did not enhance the aerodynamic performance and required a diluent for optimized performance. The impaction studies of the 50% and 75% trehalose formulations showed similar good performance to aerosolize OP. However, a significant increase (*p* value < 0.05) in performance is observed when utilizing finest trehalose particles with 75% of the drug, maximizing the amount of OP delivered such that FPD reached 5.8 mg of the calculated emitted dose 9.28 mg.

#### 3.4.2. Dispersion of 3:1 OP/Fine Trehalose Formulations

Passive DPIs devices can be grouped according to their aerodynamic resistance, where the patients’ inspiration airflow is the drive to reach the required flow rate against this resistance to aerosolize and disperse the powder efficiently. Upon forced inhalation, patients are typically able to reach a pressure drop of 4 kPa over an inhaler device, which would lead to a flow rate of 90–100 L/min in a typical low-resistance inhaler, flow rate of 40–60 L/min in a moderate-resistance inhaler, and less than 40 L/min in high-resistance inhalers [9,19,56,57]. Under this assessment, the dispersion performance is further investigated based on the variation in trehalose particle-size distribution, in addition to how this variation would affect the performance when aerosolized by the low-resistance inhaler “Aerolizer^®^” in comparison to the medium resistance inhaler “Uni-Haler”.

**Figure 9:**
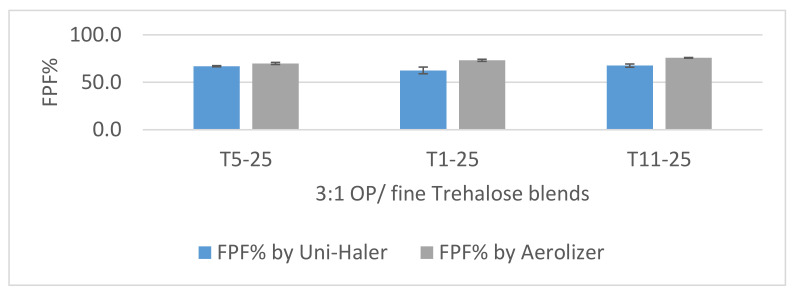
FPF% chart of 3:1 OP/fine trehalose blends using the Uni-Haler (blue bar) and Aerolizer (grey bar). Blends are arranged according to the trehalose grade from left (T5) largest, (T1) intermediate, and (T11) finest. Error bars based on calculated standard deviation from mean (*n* = 3).

Aerosolization with the UNI-Haler showed overall excellent results (≥60% FPF) as shown in Figure 9. Additionally, based on the fine variation of the trehalose particle size between the tested formulations, no significant differences (*p* value > 0.05) were observed in the resulting FPF%. The average fine-particle dose of OP achieved from the 3:1 blends was 6.2 mg. Despite the similar aerosolization performance, there was a trend of increased MMAD with the use of finer trehalose particles in blend. This observation can be related to the formed stronger agglomerates between the smaller and hence more adhesive particles. T5-25, having trehalose particles in size range 4-5 times larger than the fine OP, gave the lowest MMAD of 1.4 µm. The interaction of the fine drug with a similar sized diluent (T11) gave the largest MMAD of 1.74 µm.

The low resistance inhaler Aerolizer^®^ was used for the aerosolization performance analysis of the OP/TR formulations at a high flow rate. All three blends showed excellent dispersion with FPF ≥70%. Aerosolization at 100 L/min showed a different trend from that at 57 L/min, as the finer the total particles of both drug and diluent together, the higher the FPF%. Compared to the UNI-Haler at a lower flow rate of 57 L/min, the FPF has increased significantly (*p* value < 0.05) for all tested blends with better reproducibility as shown in Figure 9. The fine dose of OP was maximized to 7.2 mg when aerosolized by the finest trehalose (T11) at the highest flow rate tested. On the other hand, the MMAD values were similar to dispersion by the UNI-Haler, with the lowest value for T5-25 of 1.4 µm. Overall, the smaller the MMAD is, the more influential the deagglomeration. However, all the tested formulations resulted in MMAD ≤ 2 µm, which was reached under both high and medium flow rates, concluding a similar effective deagglomeration pattern.

For the dispersion mechanism of drug particles from the formulation powder mesh, several theories described the relationship between particles’ size, fluidization energy, and the deagglomeration efficiency. The “hotspot theory” as described by Staniforth JN. and experimented by Young et al. [58] and Steckel et al. [59], attributed this to the drug load that easily saturates the active sites and the fraction of the drug particles bound to the low energy sites can deagglomerate and disperse. This theory proved successful and most efficient when using ternary fine excipient (≤30 µm) that filled the active sites of the coarse diluents giving a higher probability of the low-dose drug (i.e., microgram) to bind the low energy sites for higher fine-particle fraction dispersion [60]. On the other hand, the fine-particle multiplet theory described the adhesion of the fine ternary excipient to the fine API directly forming agglomerates that are easier to disperse from the bulk course diluent [40,61]. In line with the second theory, Shur, Harris et al. have demonstrated that fine particles improve the aerosolization performance. The increased cohesive forces of diluent fine particles increase the strength of the powder bed leading to increased minimum fluidization energy; that is, when achieved by a suitable inhaler, the deagglomeration process results into a high energy collision of particles and optimum dispersion of drug fine particles [62]. The obtained results from all the aerosolization assessment can support both the hotspot hypothesis and the agglomerates hypothesis. These two hypotheses are neither inclusive nor contradicting. However, then, again, we can add that using fine diluent particles gives the capability of maximizing the deposited drug particles if using the suitable air flow for the specific minimum fluidization energy.

In our assessment, the use of the trehalose diluent as fine particles has played a different role in the mechanism of drug release from the formed agglomerates. It is proposed that the fine trehalose particles did not act as a true carrier, but as weak breaking points within the mesh of cohesive OP particles that required a high flow rate to overcome its minimum fluidization energy.

**Figure 10:**
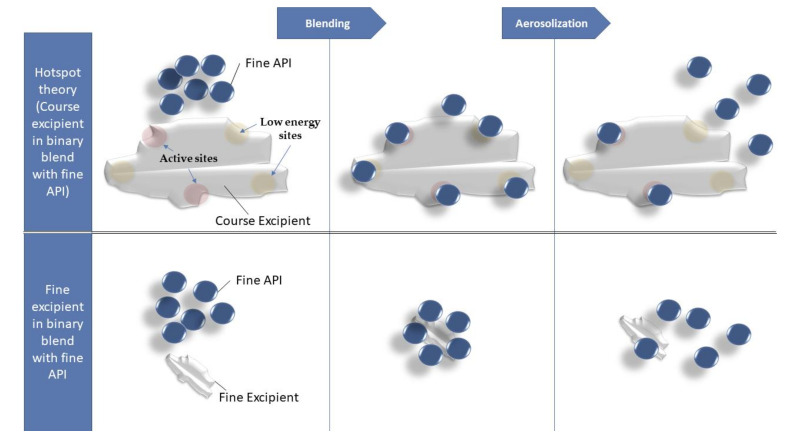
Schematic diagram of particles’ agglomeration/deagglomeration behavior upon blending/aerosolization respectively between course excipients verses fine-sized excipients in dry powder inhaler (DPI) binary blends.

Commonly the jet-milled materials with irregularly shaped particles or rough particles with asperities may limit the close contact of two particles reducing overall the van der Waals forces. It is thought that the irregular shaped fine trehalose particles reduced the van der Waals forces by being placed into the interspaces of the higher proportion of the fine rod-shaped OP particles. This reduced binding force would be achieved by introducing space in between the particles that facilitate the deagglomeration process upon sufficient airflow for aerosolization. Referring to the schematic diagram in Figure 10, the high OP dose formulation assessed, the few high-energy active sites of the fine trehalose particles would be saturated quickly, and the majority of the OP particles are binding relatively loose to the less energetic surface sites, which makes the active site theory less significant in the dispersion process. These findings are also in-line with the results of a previous study that suggested that high microporosity due to surface roughness facilitates air permeability that enhances aerosolization performance [63]. After the formulations prepared for comparison and testing various aspects as diluent types, diluent ratios, and diluent size distributions, the present work has presented the successful delivery of high-dose OP by low- and medium-resistance dry-powder inhalers. Besides, jet-milled trehalose microparticles have shown optimum physicochemical properties that promoted them as a potential excipient capable of the efficient deagglomeration process for successful aerosolization performance. The utilization of trehalose as a fine excipient acting diluent in a small ration relative to the drug has proven excellent aerosolization results in comparison to other formulations. This was in line with the recommendations of using the lowest amount of excipient to maximize the amount of API in high-dose DPI and promoting the safety on the long-term usage [64,65].

### 3.5. Cytotoxicity on Bronchial Adenocarcinoma Cells

**Figure 11:**
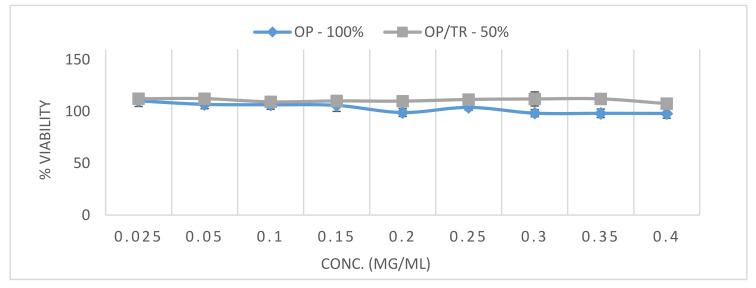
Viability percent of Calu3 cells after 4 h incubation with pure micronized OP (OP–100%) and micronized OP in combination with micronized trehalose particles in a 1:1 blend ratio (OP/TR–50%) at increasing concentrations from 0.025 up to 0.4 mg/mL for each solution. Error bars based on the calculated standard deviation from mean (*n* = 8).

To get an estimate of potential inhalation toxicity of trehalose-OP formulations, a viability assay on Calu-3 cells representing the bronchial epithelium was performed. From the viability percentage graphs obtained (Figure 11), it can be concluded that pure micronized OP (OP-100%) and micronized OP in combination with micronized trehalose particles in a 1:1 blend ratio (OP/TR-50%) did not affect viability of the cells in culture under the applied conditions. This test gives an indication that the used formulations are suitable candidates for a safe new dosage form through the inhalation route.

## 4. Conclusions

This research study demonstrated that trehalose microparticulates produced by jet milling were successfully able to aerosolize OP with positive results of a high and reasonable fine particle dose. Fine trehalose did not act as an actual carrier for the administered high dose OP; instead, the fine trehalose particles acted as linkers/spacers for the drug–particles controlled agglomeration upon preparation and facilitating deagglomeration effectively upon inhalation. A more in-depth surface energy investigation might help understand the behavior of particles acting as spacers/linkers in DPI formulations. In conclusion, two main objectives were served: the successful delivery of high dose oseltamivir phosphate for direct targeting of influenza virus in the lungs with indicated safety and the potential use of finely micronized trehalose as an inert excipient acting diluent for hydrophilic drugs to be delivered by medium- to low-resistance dry-powder inhalers. The research results presented in this study provide possible formulation routes for a potentially quick and effective delivery system in the therapeutic application of antiviral drugs required in the treatment of viral pneumonia infections.

## Figures and Tables

**Table 1:** List of all excipients’ grades (micronized or milled) showing particle size distribution as measured by laser diffraction and set in different blending ratios with micronized Oseltamivir phosphate (OP) (*n* ≥ 3, mean ± SD).

Excipient/Diluent	Diluent Grade	Fine Diluent’s Particle Size Distribution			Blend Ratio OP/Diluent	Micronized OP Formulations with Diluent
		X_10_	X_50_	X_90_		
Glucose	Micronized	1.19 ± 0.02	5.46 ± 0.21	21.32 ± 0.67	1:1	G1-50
	Milled	3.79 ± 0.21	20.23 ± 0.72	39.2 ± 0.93		G2-50
Lactose	Micronized (LH300)	0.84 ± 0.01	4.43 ± 0.03	7.71 ± 0.31		L1-50
	Milled (ML006)	1.71 ± 0.02	18.1 ± 0.37	46.14 ± 1.1		L2-50
Mannitol	Micronized	0.77 ± 0.02	2.26 ± 0.03	6.42 ± 0.23		M1-50
	Milled	1.7 ± 0.04	16.7 ± 0.49	46.35 ± 1.21		M2-50
Trehalose	Micronized 1	0.67 ± 0.01	2.23 ± 0.07	6.5 ± 0.27		T1-50
					1:3	T1-75
					3:1	T1-25
	Micronized 2	0.57 ± 0.01	1.68 ± 0.02	3.96 ± 0.11		T11-25
	Micronized 3	0.99 ± 0.02	5.26 ± 0.12	20.3 ± 0.97		T5-25
	Milled	1.51 ± 0.07	14.97 ± 0.73	40.15 ± 5.09	1:1	T2-50
					1:3	T2-75
					3:1	T2-25

**Table 2:** Fluidization energy and specific surface area measured for trehalose micronized fine grades. (*n* ≥ 3, Mean ±SD).

Trehalose Micronized Grades	Fluidization Energy (mJ)	Specific Surface Area (m^2^/g)
T5	51.1 ± 2.4	1.25 ± 0.04
T1	93.9 ± 2.0	2.36 ± 0.01
T11 (finest)	136.2 ± 2.6	2.73 ± 0.01

**Table 3:** Three-way ANOVA tabular results for each source of variation individually and combined.

Source of Variation		Sum of Squares (SS)	Degrees of Freedom (DF)	Mean Square (MS)	*p*-Value *	Significant? (Yes/No)
Carrier type	Lactose, Mannitol, Trehalose, Glucose	25.21	3	8.402	=0.0458	Yes
Carrier size	Micronized, Milled	35.88	1	35.88	=0.0012	Yes
Inhaler device	Uni-haler, Rack	1734	1	1734	<0.0001	Yes
Carrier type × Carrier size		16.37	3	5.456	=0.1435	No
Carrier type × Inhaler device		196.4	3	65.47	<0.0001	Yes
Carrier size × Inhaler device		145.3	2	145.3	<0.0001	Yes
Carrier type × Carrier size × Inhaler device		197.5	3	65.85	<0.0001	Yes
Residual		90.15	32	2.817		

* Alpha significance level set to 0.05.
